# Tamoxifen in Duchenne muscular dystrophy (TAMDMD): study protocol for a multicenter, randomized, placebo-controlled, double-blind phase 3 trial

**DOI:** 10.1186/s13063-019-3740-6

**Published:** 2019-11-21

**Authors:** Sara Nagy, Patricia Hafner, Simone Schmidt, Daniela Rubino-Nacht, Sabine Schädelin, Oliver Bieri, Dirk Fischer

**Affiliations:** 1 Division of Developmental- and Neuropaediatrics, University Children’s Hospital Basel (UKBB), University of Basel, Spitalstrasse 33, Postfach, 4031 Basel, Switzerland; 2 Department of Neurology, University Hospital Basel, University of Basel, Petersgraben 4, 4031 Basel, Switzerland; 3 Clinical Trial Unit, University of Basel, Schanzenstrasse 55, 4056 Basel, Switzerland; 4 Department of Radiology, Division of Radiological Physics, University Hospital Basel, University of Basel, Petersgraben 4, 4031 Basel, Switzerland

**Keywords:** Duchenne muscular dystrophy, Tamoxifen, Motor function measure, 6-min walk test, Quantitative muscle MRI, Randomized placebo-controlled trial

## Abstract

**Background:**

Duchenne muscular dystrophy (DMD) is an inherited neuromuscular disorder of childhood with a devastating disease course. Several targeted gene therapies and molecular approaches have been or are currently being tested in clinical trials; however, a causative therapy is still not available and best supportive care is limited to oral glucocorticoids with numerous long-term side effects. Tamoxifen is a selective estrogen receptor regulator, and shows antioxidant actions and regulatory roles in the calcium homeostasis besides its antitumor activity. In a mouse model of DMD, oral tamoxifen significantly improved muscle strength and reduced muscle fatigue. This multicenter, randomized, double-blind, placebo-controlled phase III trial aims to demonstrate safety and efficacy of tamoxifen over placebo in pediatric patients with DMD. After completion of the double-blind phase, an open-label extension of the study will be offered to all participants.

**Methods/design:**

At least 71 ambulant and up to 20 nonambulant patients with DMD are planned to be enrolled at multiple European sites. Patients will be randomly assigned to receive either tamoxifen 20 mg or placebo daily over 48 weeks. In the open-label extension phase, all patients will be offered tamoxifen for a further 48 weeks. The primary endpoint of the double-blind phase is defined as the change of the D1 domain of the motor function measure in ambulant patients or a change of the D2 domain in nonambulant patients under tamoxifen compared to placebo. Secondary outcome measures include change in timed function tests, quantitative muscle testing, and quantitative magnetic resonance imaging of thigh muscles. Laboratory analyses including biomarkers of tamoxifen metabolism and muscle dystrophy will also be assessed.

**Discussion:**

The aim of the study is to investigate whether tamoxifen can reduce disease progression in ambulant and nonambulant patients with DMD over 48 weeks. Motor function measures comprise the primary endpoint, whereas further clinical and radiological assessments and laboratory biomarkers are performed to provide more data on safety and efficacy. An adjacent open-label extension phase is planned to test if earlier initiation of the treatment with tamoxifen (verum arm of double-blind phase) compared to a delayed start can reduce disease progression more efficiently.

**Trial registration:**

ClinicalTrials.gov, NCT03354039. Registered on 27 November 2017.

## Background

Duchenne muscular dystrophy (DMD) is the most common neuromuscular disorder in childhood with an X-linked inheritance and an incidence of up to 1 in 5000 males [1, 2]. The severe and progressive weakness of skeletal muscles leads to loss of ambulation in 22% to 56% of the cases, whereas the concomitant impairment of cardiac and respiratory muscles accounts for an early mortality [2].

The clinical and pathological phenotype of DMD is caused by mutations in the dystrophin gene, which results in the total loss of dystrophin protein expression in the muscle cells [3]. Dystrophin has a major role in sarcolemmal stabilization by linking the internal actin cytoskeleton to the extracellular matrix. The protein is part of the dystrophin-associated protein complex (DAPC) including numerous integral and peripheral membrane proteins [4]. The lack of dystrophin leads to destabilization of the DAPC and to increased vulnerability and leakage of the cell membrane [5]. There is also growing evidence that the DAPC has a role in intracellular signaling pathways due to its association with several kinases, phosphatases, ion channels, receptors and transporters, pathways which seem to have significance in the disease pathogenesis [5]. If dystrophin is missing, its downstream processing is dysregulated, leading to increased Ca^2+^ influx, oxidative stress and mitochondrial dysfunction [6]. An increased cytosolic Ca^2+^ level is a result of many dysfunctional pathways involving store-operated channels, stretch-activated channels, the sarcolemmal Ca^2+^ pump and calpains [5, 7, 8]. Increased intracellular Ca^2+^ levels were shown in mdx mice and also in muscle biopsies from DMD patients [9–12], implicating Ca^2+^ as a main driver of the pathology responsible for apoptosis and necrosis. It has even been shown that increased Ca^2+^ influx alone is sufficient to induce muscular dystrophy through transient receptor potential canonical 3-mediated pathways [13].

Several targeted gene therapies and molecular approaches counteracting the dysfunctional intracellular pathways have been tested in *mdx* animal models and many are currently investigated in clinical trials [6, 14]. However, the proof of principle for DMD patients has yet to be established. Tamoxifen is a selective estrogen receptor regulator and its use is well established in patients with breast cancer [15]. Tamoxifen acts as an agonist or antagonist of estrogen in a tissue-dependent manner. Advantages of tamoxifen include its antioxidant actions and regulatory roles in calcium homeostasis [16–18]. Based on preliminary data provided by the investigator, tamoxifen leads to an elevated level of pro-inflammatory cytokines and growth factors involved in muscle regeneration and fibrosis (transforming growth factor-β (TGFβ), insulin-like growth factor 1 (IGF1) and osteopontin) and to an increased capacity of muscle-purified mitochondria to buffer cytosolic calcium. Tamoxifen is able to prevent bone loss and has been shown to increase the height of short boys by decreasing the rate of bone maturation [19, 20]. In a mouse model of DMD, oral tamoxifen stabilized the membrane of myofibers, significantly improved muscle strength, reduced muscle fatigue, and slowed phenotype [21, 22]. Furthermore, tamoxifen could reduce fibrosis of the heart muscle and diaphragm by about 50%. The effectiveness of tamoxifen has recently been shown in another fatal congenital muscular disorder. In a mouse model of myotubular myopathy, tamoxifen could improve force, decrease disease progression and prolong survival [23, 24].

Preclinical and clinical data for tamoxifen in muscular dystrophy are promising and the findings suggest its usage also in patients with DMD. According to yet unpublished preliminary results of an open-label trial (ClinicalTrials.gov identifier NCT02835079), tamoxifen has beneficial effects at a daily dose of 20 mg. Based on personal communication with the investigator, patients with DMD taking tamoxifen remained stable over an observation time of 12 month as assessed by North Star Ambulatory Assessment (NSAA) and timed function tests (TFT) including the 6-min walk test (6MWT). Furthermore, all patients showed good tolerance of the medication without any treatment-related serious adverse events. Tamoxifen has also been previously tested in the pediatric population for low- and high-grade glioma, desmoid tumor, pubertal gynecomastia and short height [25–28]. Treatment with tamoxifen was well tolerated in each study, even when used at higher doses.

The purpose of this study is to evaluate the effect of tamoxifen on muscle function and muscle force compared to placebo in ambulant and nonambulant children with DMD. Furthermore, regular pharmacokinetic evaluation of tamoxifen plasma levels and metabolites, and detailed analysis of biomarkers of muscle degeneration, muscle necrosis (e.g., creatine kinase (CK) and alkaline phosphatase (AP)) and muscle dystrophy (e.g., tumor necrosis factor (TNF), TGFβ, interleukin (IL)-1β and IL-6) could provide a better understanding of the mode of action of tamoxifen. With the current lack of an effective and safe long-term treatment for DMD patients, tamoxifen could be a milestone in improving clinical outcome while providing good safety and tolerability.

## Methods/design

### Study design

This is an investigator-initiated, multicenter, randomized, double-blind, placebo-controlled phase III efficacy and safety trial in patients with DMD. This part of the study is conducted over 48 weeks. The two treatment arms include tamoxifen (verum) and placebo (control). We plan to enroll at least 71 ambulant patients aged between 6.5 and 12 years (group A) and 16–20 nonambulant patients aged between 10 and 16 years (group B) at multiple European sites (Belgium, France, Germany, Netherlands, Spain, Switzerland, Turkey, and the United Kingdom). After completion of the double-blind phase, all participants will be offered the option of taking part in an open-label extension (OLE) trial of the main study. In this part, all patients will receive tamoxifen for 48 weeks.

**Fig. 1:**
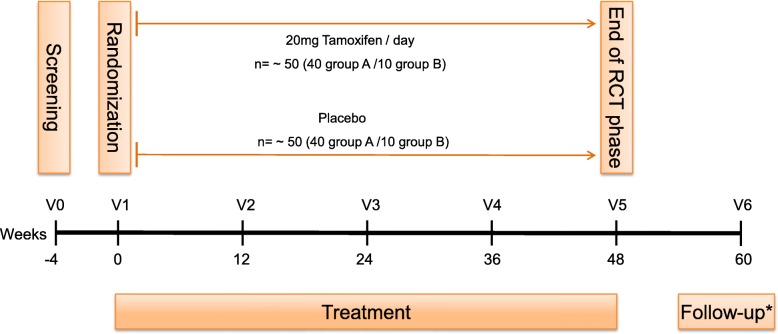
Flow chart showing the study design of the placebo-controlled, double-blind phase of the tamoxifen in Duchenne muscular dystrophy (TAMDMD) study. *For those patients who do not enter the open-label extension phase. RCT randomized controlled trial, V visit

**Fig. 2:**
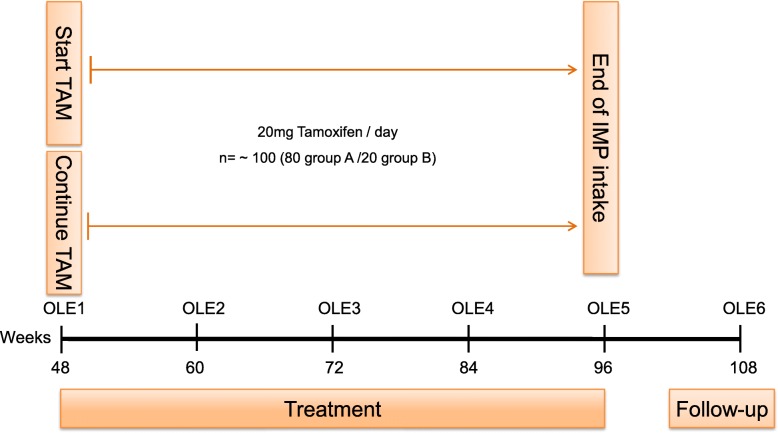
Flow chart showing the study design of the open-label extension phase (OLE) of the tamoxifen in Duchenne muscular dystrophy (TAMDMD) study. IMP investigational medicinal product, TAM tamoxifen

The trial was approved by the local ethics committee (ethics committee of both Basel cantons, 2017–01708), the National Swiss Drug Agency (Swissmedic, 2018DR3068), the European Union Drug Regulating Authorities Clinical Trials (EudraCT 2017–004554-42), and the European Union Drug Regulating Authorities Pharmacovigilance (EudraVigilance). The study was registered at ClinicalTrials.gov (NCT03354039) and at the Swiss National Clinical Trials Portal (SNCTP000002387) prior to recruitment. The SPIRIT flow chart showing the main study design is seen in Fig. 1, while Fig. 2 represents the study design of the OLE phase. The Recommendations for Interventional Trials (SPIRIT) checklist on which the study protocol is based is presented as Additional file 1.

### Inclusion criteria

Only male patients with a molecular diagnosis of DMD are included in the study. Ambulant patients in group A must fulfill the following criteria at screening: 6.5 to 12 years of age, weight >20 kg, stable treatment with glucocorticoids >6 months, ability to walk at least 350 m without assistance in 6MWT and a D1 domain of the motor function measure (MFM) >40%. Nonambulant patients in group B must be between 10 and 16 years of age at time of screening, be off glucocorticoids for >6 months, and have no ability to walk more than 10 m. Patients taking ataluren should be under a stable ataluren treatment for at least 3 months or be off ataluren treatment for at least 3 months before screening. For participation in the OLE trial, a preceding completion of the main study is mandatory.

### Exclusion criteria

Patients fulfilling the following criteria are excluded from the study: females, allergy to tamoxifen, use of tamoxifen or testosterone within the last 3 months, known or suspected malignancy, clinically relevant disease with limitation of renal, liver or heart function, injury impacting functional testing, planned or expected spinal fusion surgery during the study period, previous spinal fusion surgery within the last 6 months, galactosemia, congenital lack of lactase, and glucose-galactose malabsorption. Patients taking CYP2D6 inhibitors, CYP3A4 inducers, platelet aggregation inhibitors, coumarin-type anticoagulants, or drugs metabolized by CYP2C9 must also be excluded. Furthermore, patients with certain eye disorders (cataract, retinopathy, optic neuropathy, alteration of the cornea) and those with laboratory abnormalities such as anemia, thrombocytopenia, leukopenia, neutropenia or agranulocytosis are also not allowed to be included. Patients taking part in the trial should not concomitantly participate in any other interventional trial or have done up to 3 months prior to screening.

### Randomization and blinding

Patients who meet the study admission criteria and do not fulfill any exclusion criteria are enrolled and randomly assigned to either the active treatment or the control treatment with a treatment allocation of 1:1. The study is double-blind; therefore, all clinical investigators and all patients and their caregivers will remain blinded throughout the trial. Patients who withdraw from the study will not be replaced. At the end of the main trial all patients will be asked to join the OLE trial, receiving active treatment only. Participants can leave the study at any time and for any reason.

### Intervention

All patients who undergo randomization will receive an oral drug once daily: either tamoxifen at a dose of 20 mg (verum) or placebo (control). The placebo medication will have the same texture, taste and color as the interventional drug, but without any active ingredients. Both the interventional and the control drug are manufactured by Hexal AG, Industriestrasse 25, 83,607 Holzkirchen, Germany. Tamoxifen tablets will be protected from light and moisture.

The intervention period lasts 48 weeks with an end-of-study visit at week 60. The study medication is taken on top of standard care with glucocorticoids in group A if treatment with steroids has been stable over the last 6 months prior to screening (dose adaptations according to weight change are allowed). After completing the main study, an OLE is performed where all patients will be offered to receive tamoxifen.

### Study procedure

Patients enrolled in the study attend a screening visit 4 weeks prior to baseline. Study visits take place every 12 weeks during the intervention period of 48 weeks. The double-blind phase of the study ends with a visit at week 48, with a follow-up visit at week 60 for patients not participating in the extension phase.

At screening (visit 0), patients and their caregivers receive detailed information about preclinical data, the study procedure and possible benefits and risks of the trial. After signing the informed consent form, inclusion and exclusion criteria will be verified. Patients/caregivers are asked to sign a separate optional informed consent form for collection of data for further genetic and biomarker analysis. Patients fulfilling the criteria will be enrolled and undergo the following investigations: physical examination, vital signs, blood draw for safety laboratory tests, and physiotherapy assessment including MFM, NSAA, proximal upper limb function (PUL), TFT (6MWT, 10-meter walk/run test (10MWT), time to rise from the floor), and quantitative muscle testing (QMT) using grip force. An ophthalmological examination including visual acuity and slit lamp is performed either at screening or in the time period between screening and baseline.

The baseline visit (visit 1) takes place no longer than 4 weeks after screening. During this visit, fulfillment of the inclusion/exclusion criteria are reassessed and qualified patients randomized to receive the study medication (verum or placebo). During this visit, the following procedures are performed: physical examination, vital signs, Tanner staging (assessment of the external primary and secondary sex characteristics), blood draw, adverse events, calculation of Wells score (risk estimation for deep vein thrombosis), complete physiotherapy assessment as described above, quantitative magnetic resonance imaging (qMRI) of the thigh muscle measuring the mean fat fraction (FF) and T2 relaxation time (T2), ophthalmological examination, patient-reported outcome measures using the Personal-Adjustment and Role Skills Scale (PARS-III) and Raven’s Coloured Progressive Matrices (RCPM). In selected sites, an x-ray for bone age determination and dual-energy x-ray absorptiometry (DEXA) will be performed during this visit.

Six weeks after baseline, patients receive a telephone call to capture any early adverse events and to assess the Wells score.

At weeks 12 and 36 (visits 2 and 4, respectively) the following procedures will be performed: physical examination, vital signs, adverse events, blood draw, Wells score, and complete physiotherapy assessment.

At weeks 24 and 48 (visits 3 and 5, respectively) the following procedures will be performed: physical examination, vital signs, Tanner staging, blood draw, Wells score, complete physiotherapy assessment, qMRI, ophthalmological examination, and PARS-III. At week 48, RCPM will be repeated and, in selected sites, x-ray bone age determination and DEXA will be performed. For patients not participating in the OLE phase, a follow-up visit takes place at week 60 (visit 6) and includes the following: physical examination, vital signs, adverse events, blood draw, and Wells score.

Informed consent for the OLE phase will be offered to all patients/caregivers already at screening, but withdrawal from the extension phase is possible at any time throughout the study. Patients confirming their participation in the OLE phase will be re-checked for the inclusion and exclusion criteria at week 48 (1OLE). All patients will receive a telephone call 6 weeks after starting the OLE and are asked for early adverse events and to evaluate the Wells score. Study visits will be performed every 12 weeks and include the same assessments as the main study. At weeks 60 (2OLE) and 84 (4OLE) the following procedures will be performed: physical examination, vital signs, adverse events, blood draw, Wells score, and complete physiotherapy assessment. At weeks 72 (3OLE) and 96 (5OLE) the following procedures will be performed: physical examination, vital signs, Tanner staging, blood draw, Wells score, complete physiotherapy assessment, qMRI, ophthalmological examination, and PARS-III. During 5OLE, RCPM will be repeated and, in selected sites, x-ray bone age determination and DEXA will be performed. For all patients in the OLE, a follow-up visit at week 102 (6OLE) is planned including physical examination, vital signs, adverse events, blood draw and Wells score. Tables 1 and 2 show the detailed schedule of the placebo-controlled, double-blind randomized controlled trial and the OLE phase of the study, respectively.

**Table 1:**
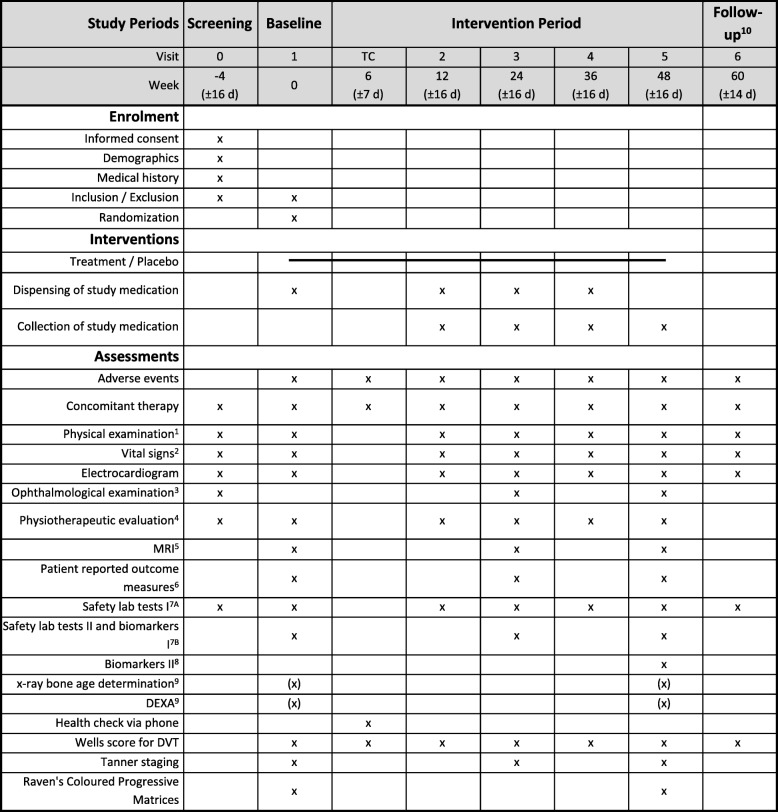
Detailed schedule of the placebo-controlled, double-blind phase of the tamoxifen in Duchenne muscular dystrophy (TAMDMD) study

^1^General pediatric physical examination, including anthropometric measurements (weight and height). ^2^Blood pressure and heart rate. ^3^Including visual acuity and slit-lamp examination. ^4^Motor function measurement, North Star Ambulatory Assessment, proximal upper limb function, timed function tests including 6-min walk test, 10-min walk/run test, supine up time, and quantitative muscle testing using grip force. ^5^Thigh muscle fat fraction and T2 relaxation time on quantitative magnetic resonance imaging (MRI). ^6^Personal-Adjustment and Role Skills Scale. ^7A^Chemistry: creatine kinase, gamma GT, total bilirubin, alkaline phosphatase, creatinine, electrolytes (Na^+^, K^+^, Ca^2+^), urea, triglycerides; hematology: full blood count for erythrocytes, leukocytes (with a differential), platelets, hemoglobin, hematocrit, absolute neutrophil count. ^7B^Sex hormone function (luteinizing hormone, follicle-stimulating hormone, testosterone, sex hormone binding globulin, alpha-fetoprotein), preservation of serum and EDTA full blood to be able to measure plasma levels of tamoxifen and its metabolites (endoxifen and 4-OH-tamoxifen) and biomarkers I (connective tissue growth factor, fibroblast growth factor 21, insulin growth factor 1, interleukin (IL)-1β, IL-6, matrix metalloproteinase (MMP)-2, MMP-9, osteopontin, platelet-derived growth factor (PDGF)-A, PDGF-B, tissue inhibitor of metalloproteinases 1, transforming growth factor-β, tumor necrosis factor. ^8^Biomarkers II: Cyp2D6, Cyp3A4. ^9^In selected sites only. ^10^Only for patients not participating in the open-label extension phase. d day, DEXA dual-energy x-ray absorptiometry, DVT deep vein thrombosis, TC telephone call

**Table 2:**
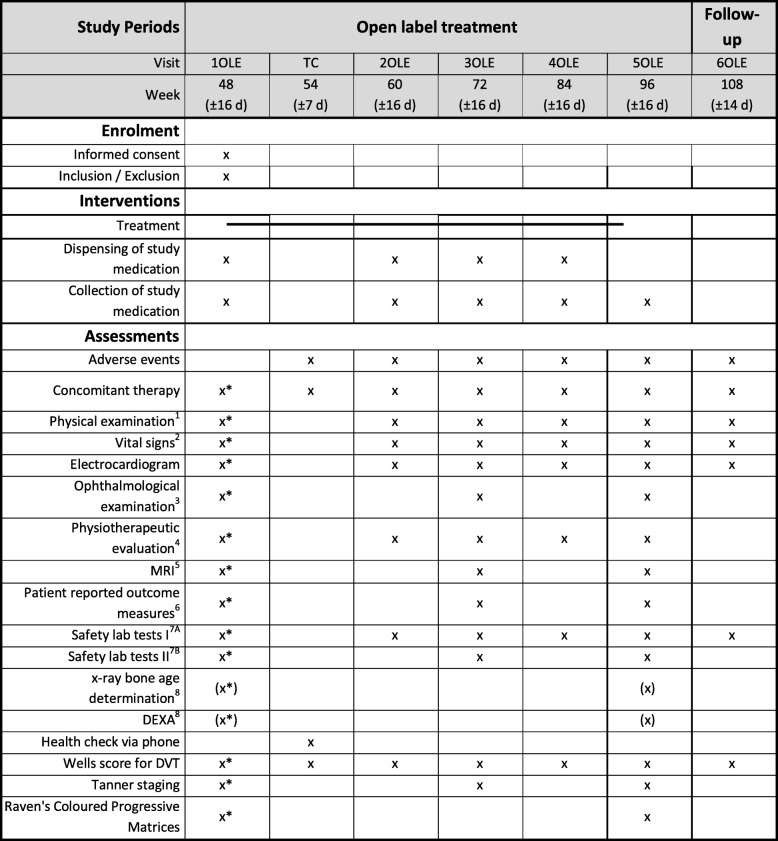
Detailed schedule of the open-label extension phase (OLE) of the tamoxifen in Duchenne muscular dystrophy (TAMDMD) study

^1^General pediatric physical examination, including anthropometric measurements (weight and height). ^2^Blood pressure and heart rate. ^3^Including visual acuity and slit-lamp examination. ^4^Motor function measurement, North Star Ambulatory Assessment, proximal upper limb function, timed function tests including 6-min walk test, 10-min walk/run test, supine up time, and quantitative muscle testing using grip force. ^5^Thigh muscle fat fraction and T2 relaxation time on quantitative magnetic resonance imaging (MRI). ^6^Personal-Adjustment and Role Skills Scale. ^7A^Chemistry: creatine kinase, gamma GT, total bilirubin, alkaline phosphatase, creatinine, electrolytes (Na^+^, K^+^, Ca^2+^), urea, triglycerides; hematology: full blood count for erythrocytes, leukocytes (with a differential), platelets, hemoglobin, hematocrit, absolute neutrophil count. ^7B^Sex hormone function (luteinizing hormone, follicle-stimulating hormone, testosterone, sex hormone binding globulin, alpha-fetoprotein). ^8^In selected sites only. *Will be assessed at visit 5 (end of study visit). d day, DEXA dual-energy x-ray absorptiometry, DVT deep vein thrombosis, TC telephone call

### Quality assurance

This study will be conducted in compliance with the protocol, the current version of the Declaration of Helsinki, the Good Clinical Practice from the International Council for Harmonization (ICH-GCP) and with all national legal and regulatory requirements. To achieve this the members of the study team are required to hold an updated certification in GCP. All physiotherapists will be trained and certified for motor function measurements. To control adherence to the intervention, a qualified person from the study team will check the number of dispensed/taken medications and complete a study-specific drug accountability form at each visit. A distributor (Thermo Fisher Scientific, Allschwil, Switzerland), who is not involved in the study, will pack, label and dispense the medication according to the randomization procedure to prevent unblinding of the investigators.

### Safety assessments

Adverse events will be monitored throughout the study. At every visit patients and their caregivers will be asked about adverse events. Measurement of vital parameters (blood pressure, heart rate, electrocardiogram), physical examination, and assessment of the Wells score will be performed. Safety laboratory measurements include full blood count, CK, AP, gamma-glutamyl transferase (gamma GT), total bilirubin, urea, creatinine, electrolytes, and triglyceride levels. Further laboratory assessments (such as luteinizing hormone (LH), follicle-stimulating hormone (FSH), testosterone, sex hormone binding globulin (SHBG), alpha-fetoprotein (AFP), plasma levels of tamoxifen and its metabolites, and biomarkers) will be performed at defined time points during the study (Figs. 1 and 2). Visual acuity and split-lamp examinations performed by an ophthalmologist or optician and Tanner staging are performed at three study visits both during the intervention and the OLE phase. RCPM will be performed at two time points in both the intervention and the OLE phases. X-ray bone age determination and DEXA scan are planned only in selected sites. A safety follow-up will take place for patients with early study termination and include the following: assessment of adverse events, vital parameters, physical examination, safety blood test and biomarkers, Tanner staging, Wells score, ophthalmological examination, RCPM, physiotherapeutic evaluation and DEXA scan at selected sites.

The study medication must be immediately discontinued in the following cases: decrease in visual acuity of more than 30% compared to baseline, cataract or optic nerve involvement or any other significant novel eye disease, thromboembolic event, newly diagnosed malignancy, or any other severe drug-related adverse events or serious adverse events. Also, laboratory abnormalities including platelet count <75 × 10^9^/l, absolute neutrophil count <1 × 10^9^/l and serum Ca^2+^ >2.6 mmol/l must lead to treatment discontinuation. Furthermore, withdrawal of consent, patient noncompliance, logistical reasons, or inability to attend study visits can also lead to early patient termination from the study. Finally, in case of negative (nonsignificant) differences of the primary endpoint after analysis of the randomized controlled trial phase, the OLE phase will be finished and the active treatment stopped immediately in all study patients.

All serious adverse events must be reported to the Sponsor-Investigator of the study within 24 h after an investigator becomes aware of such an event. In the case of life-threatening serious adverse events or those resulting in death, the local national Ethics Committee must also be informed within 7 days. Patients with adverse events will be followed up by the investigator for up to 30 days after the last visit.

Both patients and their partners of child-bearing potential must employ a reliable method of birth control during the study and for a further 90 days after the intake of the study drug. Special attention must be paid to protection from the sun throughout the trial and up to at least 12 weeks after the trial ends. In case of problems and safety concerns that cannot be solved with on-going blinded treatment, the participant’s allocated intervention will be revealed. Unblinding can be performed by the investigators using the randomizer.at tool. Any important protocol modifications will be directly communicated to all relevant parties. Under emergency circumstances, deviations from the protocol to protect the rights, safety and well-being of human subjects may proceed without prior approval of the sponsor and the ethics committee. Deviations must be documented and reported to the sponsor and the ethics committee as soon as possible.

### Efficacy outcome measures

Outcomes and endpoints are the same for both the placebo-controlled and OLE phases of the trial.

#### Primary efficacy outcome measures

##### Change of D1 domain of motor function under tamoxifen compared to placebo in ambulant patients

In group A, which includes only ambulant patients, the primary efficacy outcome is the change of motor function measured by the MFM. MFM is a commonly used assessment in patients with DMD that has been validated in Lyon, France [29–31]. The test measures all dimensions of the motor function by evaluating standing and transfer (D1 domain), axial and proximal (D2 domain), and distal motor function (D3 domain). The reliability of the MFM has been examined in several studies. It is described as being sensitive to treatment response [32] and to disease progression even within as short a period as 3 months [33, 34]. The D1 domain of the MFM, assessing standing and transfer, was shown to be the most powerful parameter to detect clinical decline compared to D2 and D3 domains [35].

##### Change of D2 domain of motor function under tamoxifen compared to placebo in nonambulant patients

Since the D1 domain remains around 0% in patients who have lost ambulation it cannot be applied to monitor disease progression or treatment response in nonambulant patients. In contrast, the D2 and D3 domains are more suitable for nonambulant patients as these assess the retained upper limb functions [29, 32, 33]. The D2 domain in particular was shown to be informative, with a decrease of 9.4% per year in nonambulant patients [33]. Therefore, the D2 domain, assessing axial and proximal motor function, is defined as the primary endpoint for the group B (nonambulant) population of the study.

#### Secondary efficacy outcome measures

##### Change of motor function measured by the MFM total score, its D2 and D3 domains, NSAA and PUL under tamoxifen compared to placebo

The total MFM and also its D2 and D3 domains are defined as secondary endpoints, giving complete information about all three dimensions of the motor function including axial, proximal and distal motor function. The NSAA is another commonly used and validated test to measure motor function in ambulant children with DMD [36, 37]. The test can be used in children from the age of 4 years [38]; moreover, a revised version of NSAA was recently proposed to be used in patients above 3 years of age [39]. The functional testing of the upper limbs is a reliable parameter both in ambulant and nonambulant patients, but its application is especially meaningful in patients who have lost ambulation [40].

##### Change in TFT measured by 6MWT, 10MWT and time to rise from the floor under tamoxifen compared to placebo

TFT are useful clinical assessments in ambulant patients since they provide important information about the patient’s endurance. The 6MWT has especially been shown to be a good surrogate marker of disease progression as it independently predicts loss of ambulation [41–44]. A walking distance of <350 m in the 6MWT is considered a strong predictor of clinical decline [41, 42, 45, 46]. TFT of the study will include 6MWT, 10MWT and time to rise from the floor.

##### Change in QMT measured by grip force under tamoxifen compared to placebo

To measure the change in isometric muscle strength under treatment compared to placebo QMT using grip force measured by a handheld dynamometer will be performed [47].

##### Change in qMRI measurement of thigh muscles using FF and T2 under tamoxifen compared to placebo

qMRI is considered to be the most sensitive biomarker in patients with DMD. It assesses disease-related tissue changes including fat replacement (muscle FF), muscle edema and inflammation (T2). It objectively correlates with functional outcome measures in detecting clinical decline [48–51], reliably shows subclinical changes, and can even predict loss of ambulation [52, 53]. In this study, qMRI of all thigh muscles (flexors, extensors, and adductors) will be performed using FF and T2.

#### Patient-reported outcome measures

PARS-III is a parent-reported assessment that gives useful information about the psychosocial adjustment of a child. The test has been used in several chronic diseases and described as reliable and valid in patients with DMD [54]. The PARS-III will be performed throughout the study (at baseline, at weeks 24, 48, and in the OLE phase at weeks 72 and 96).

#### Safety outcome measures

The following safety assessments are performed at each visit: blood pressure, heart rate, weight, height, Wells score for deep vein thrombosis and electrocardiogram. Furthermore, the following safety laboratory assessments will be performed by routine testing at each visit: CK, gamma GT, total bilirubin, urea, AP, creatinine, electrolytes, triglycerides and full blood count including erythrocytes, leukocytes (with a differential), platelets, hemoglobin, hematocrit, and absolute neutrophil count. Tanner staging, ophthalmological examination, laboratory sex hormone concentrations (LH, FSH, testosterone, SHBG), and AFP will be monitored at baseline, at weeks 24 and 48, and also in the OLE phase at weeks 72 und 96. The cognitive function in the study population will be monitored by means of the RCPM test at baseline, at week 48, and also at week 96 in the OLE phase. RCPM is a validated, easy-to-perform, language-independent assessment which can be used in patients with severe motor or cognitive impairment. Normative values are available for the patient age group. RCPM has classically been used to measure global cognitive performance and has been used in DMD [55]. In selected sites, x-ray bone age determination (examination of the left hand) and lumbar spinal bone density measured by DEXA will be performed at baseline, at week 48, and at week 96 in the OLE phase.

#### Further outcomes of interest

The following markers of tamoxifen metabolism will also be assessed. Cyp2D6 and Cyp3A4 are the enzymes involved in the degradation of tamoxifen, while endoxifen and 4-OH-tamoxifen are considered as potential active metabolites. To examine muscle dystrophy biomarkers, TNF, TGFβ, IL-1β, IL-6, platelet-derived growth factor-B, IGF1, fibroblast growth factor 21, connective tissue growth factor, osteopontin, tissue inhibitor of metalloproteinases 1, matrix metalloproteinase (MMP)-2, and MMP-9 will be analyzed.

#### Objectives of the OLE phase of the main study

Outcome measures are the same for both the placebo-controlled and OLE phases of the trial. The goal of the OLE part is to test if earlier initiation of tamoxifen treatment (verum arm of the double-blind study) can reduce disease progression more efficiently. Therefore, disease progression during the OLE phase will be compared between both OLE treatment arms. A further aim is to obtain more safety and efficacy information.

### Randomization scheme

At the baseline visit patients will be randomly assigned either to the active treatment or the control group. Stratification will be performed according to glucocorticoid use into the three strata of continuous user, intermittent user, and no user of glucocorticoid. The randomization procedure will be implemented by the Clinical Trial Unit (CTU) of the University Hospital Basel into a web-based randomization service tool for multicenter trials (randomizer.at), provided by the Institute for Medical Informatics, Statistics and Documentation of the Medical University Graz. It will include a standard minimization algorithm which will ensure that the treatment groups are balanced within each stratum. To avoid predictable alternation of treatment allocation, and thus potential loss of allocation concealment, patients will be allocated with a probability of 80% to the treatment group to minimize the difference between the groups within the patient’s stratum.

### Sample size estimation

Sample size estimation was performed for the ambulant patients to allow the identification of a significant difference in the D1 domain between the two treatment groups (verum versus placebo). A type I error rate of α = 0.05 was used and the power was set at 1 – β = 0.8. The sample size estimation was based on clinical data from patients in the placebo arm of the Treatment with L-citrulline and metformin in Duchenne muscular dystrophy (DMD02) study [52]. The mean change in D1 domain of these patients was 9.1% during 25 weeks while, previously, an annual D1 decline of 17.2% was reported in ambulant DMD patients [32].

A semiparametric method was performed to account both nonparametrically for the available dataset and parametrically for the treatment shift. Each sample size, *n*i = 1, ..., 50 = 30, ..., 1500, was evaluated by drawing 99 times an individual dataset of size *n*i from the pilot study dataset. Here, *n*i patients were sampled with replacement from a pilot dataset. For each sample, the average disease progression (mean) between baseline and follow-up was estimated. For patients assigned to placebo, the follow-up measurement of the pilot data was used as the expected follow-up measurement. In patients assigned to tamoxifen, the follow-up measurement was recalculated by adding the treatment effect. An analysis of covariance (ANCOVA) model was used to assess whether the D1 domain significantly differed between the two treatment groups after 48 weeks when adjusting for baseline values. Based on the assumption that tamoxifen reduces the mean decrease of D1 by 50% within 1 year, 79 patients should be screened in group A of the study population. A reduction of 50% corresponds to an average loss of 4.5 points in the D1 subscore under verum compared to a loss of 9 points under placebo. Assuming a screening failure rate of 10% and a drop-out rate of 15%, 79 screened patients would result in 71 randomized patients to ensure 60 evaluable patients in total.

Sample size estimation for the nonambulant patient group was based on practical considerations. Group B does not constitute our main study population and is included to obtain some additional safety and efficacy data. Therefore, statistical power calculations were omitted in this population.

### Statistical analysis

#### Primary analysis

The primary analysis will be based on the D1 domain of the MFM in ambulant patients. For both groups A and B, two analysis sets will be built. The full analysis set will include all patients who had at least one follow-up measurement at week 24 or later. The per-protocol set will include all patients from the full analysis set who had the 1-year follow-up visit, and who had a drug compliance of 70–100%. The analysis will be done on the intention-to-treat principle. The measurement at randomization (week 0) will be considered as the baseline and the measurement at week 48 as the primary endpoint. An ANCOVA approach will be used: D1 at 48 weeks will be modeled using a linear regression model including D1 at baseline and treatment groups (verum versus placebo) as predictors in the model. Medication with glucocorticoids will be included as a covariable. Since D1 can be performed only in ambulant patients, this patient group will be used for the primary analysis. The model will be controlled by checking the model assumptions and by inspecting the residuals and leverages.

A following sensitivity analysis will be done to support the main analysis: concomitant use of CYP2D6 inhibitors in the treatment arm as an interaction term, glucocorticoid use in the treatment arm as an interaction term, exclusion of adjustment for glucocorticoid use, exclusion of multiple imputations (the last observation available at least 24 weeks after randomization will be imputed for missing values), inclusion of all measurements at weeks 0, 12, 24, 36, and 48 in a mixed -effects model, and repetition of the analysis on the per-protocol dataset.

For further efficacy analysis, the previously described ANCOVA approach (based on the primary outcome measure) will be performed also for the OLE phase.

#### Secondary analyses

The goal of the secondary analyses is to show superiority of tamoxifen compared to placebo in regard to the secondary objectives. The two patient groups will be analyzed in separate models for all endpoints. The analyses will be performed on the full analysis set and include the following: MFM total score, a subset of D1 domain scores showing a more distinct decrease during disease progression in several datasets, D2 domain, D3 domain, NSAA, TFT, PUL, QMT, qMRI using muscle FF, and PARS-III. The analyses will use the same statistical model as specified for the main analysis; however, in the calculation of the muscle FF, a beta regression model will be performed.

For safety reasons, an interim analysis is planned after the completion of the double-blind phase of the study. The number and type of treatment-emergent as well as treatment-related adverse events will be summarized and compared between treatment groups. A safety analysis will be performed similarly also for the OLE phase.

### Quality control and data protection

The clinical study can only begin in a certain country once approval from all required authorities in that particular country has been received. Any additional requirements imposed by the authorities shall be implemented. The safety of the study will be assured by an independent safety monitoring board organized by the CTU of the University Hospital Basel, Switzerland. It will be composed of independent experts to protect the patient’s safety and will also include Duchenne patient organization representatives. An unblinded, independent statistician will be involved. Study monitoring will be performed by the Clinical Research Organization (CRO) multi -service-monitoring (Maxhüttenstrasse 11, 93,055 Regensburg, Germany). The monitor will be responsible for controlling the inclusion and exclusion criteria, all patient data, occurrence of serious adverse events, and drug accountability. The CRO SCRATCH Pharmacovigilance GmbH (Schlossstrasse 25, 35,510 Butzbach, Germany) will be responsible for serious adverse event processing and keeping the safety database.

Direct access to source documents will be permitted for purposes of monitoring, audits and inspections. The investigators of the study will have access to the protocol and the datasets. The statistician will have access to the statistical code during and after the study. A transfer of data will only take place for study purposes and only in encoded form. For inspection purposes, insight to source data will be permitted to the member of the appropriate authorities and also for members of the local ethics committee. During the study, confidentiality will be guaranteed. The principal investigator will guarantee compliance with national and international data security.

### Storage of biological material and related health data

Biological material and related health data will be stored in an encrypted format for follow-up analyses. Blood samples for biomarker analyses will be stored under standardized conditions at each study center until the last patient visit. Samples will then be sent to the laboratories in Lausanne, Switzerland, to perform the planned, batch-wise analyses.

## Discussion

DMD is a devastating genetic neuromuscular disorder that affects children from their early years of life. Due to their known anti-inflammatory effects, glucocorticoids have been used since the 1980s as a palliative treatment, and their regular intake has been linked to improved muscle strength, muscle function, and even prolonged ambulation [55]. Despite their recognizable advantages in DMD, the extensive side effects of long-term corticosteroid use including weight gain and the high risk for osteoporosis and diabetes mellitus make this therapy option less attractive in patients at a young age. The improved understanding of disease pathophysiological mechanisms led to the initiation of new therapy approaches with the hope of seeing more significant treatment effects combined with better safety. However, most of the novel therapeutic approaches, such as exon skipping therapies or “read through” approaches for mutations, are often applicable to a limited number of patients only because of their mutation-specific functioning.

Tamoxifen, an estrogen receptor modulator used in estrogen receptor-positive breast cancer, is a promising agent and seems to have the potential to reduce disease progression in DMD. Based on encouraging preclinical research data conducted in a mouse model of DMD [21, 22], we aim to test the safety and efficacy of tamoxifen in patients with DMD. Tamoxifen has already been tested in children with brain tumors, pubertal gynecomastia or short height, and showed good safety and tolerability [25–28].

This multicenter, randomized, double-blind and placebo-controlled trial investigates whether tamoxifen can reduce disease progression by at least 50% compared to placebo in 6.5–12-year-old ambulant (group A) DMD patients. The goal of the OLE part is to test if earlier initiation of tamoxifen treatment (patients of the verum arm of the randomized controlled trial phase of this study) can reduce disease progression more efficiently than later treatment onset. Therefore, disease progression during the OLE phase will be compared between both OLE treatment arms. Finally, we also address the question whether tamoxifen can reduce clinical decline in 10–16-year-old nonambulant DMD patients who are not receiving steroids (group B). The inclusion of these patients was highly encouraged by the European Medicines Agency (EMA) in order to obtain more data on safety and efficacy in this patient group, even if statistical significance is not to be expected. The design of this trial, including duration, inclusion and exclusion criteria, efficacy and safety endpoints and statistical analysis regarding sample size and final analysis, have been discussed in detail and agreed with external opinions (Committee for Medicinal Products for Human Use (CHMP) of the EMA, Advisory Committee for Therapeutics (TACT) of Translational Research in Europe for the Assessment and Treatment of Neuromuscular Disease (TREAT-NMD), Sandoz, who are providing the investigational medicinal product, national competent authorities, and responsible ethical committees of the participating countries).

The primary outcome of the study is defined by the MFM, including evaluation of standing and transfer, axial, proximal, and distal motor function. TFT, particularly the 6MWT, are commonly used endpoints in clinical trials; however, most of them can only be performed in ambulant patients and show age dependency due to changes in motor development [41, 42]. According to as yet unpublished data from our group, sample sizes showing a longitudinal treatment response of at least 50% are the lowest if the D1 domain of the MFM or MFM total score is used compared to use of TFT including the 6MWT. MFM is commonly used in the field of neuromuscular disorders and is validated in patients with DMD [21, 22]. MFM is not only a sensitive test with a low inter-rater and intrarater variability, but it also meets a highly important criterion of outcome measures, namely the sensitive prediction of clinical decline and loss of ambulation. Total MFM score of 70% has been described to be predictive for loss of ambulation in 1 year [33]; however, in ambulant patients, the D1 domain seems to be of higher relevance [33, 34]. A D1 domain of 40% or a mean yearly reduction of 17.2% has been described to be predictive for loss of ambulation [33]. Therefore, D1 was chosen to be the primary endpoint for ambulant patients included in the study. In those patients who have already lost ambulation and are therefore not able to perform the test, the assessment of axial and proximal motor function (D2 domain) gives sensitive information about clinical decline [33]. According to this, the D2 domain will be used as the primary endpoint for the nonambulant population of the study. Further clinical outcome measures, including TFT, NSAA and QMT, will be evaluated as secondary endpoints.

Since none of the clinical tests are completely independent of the evaluator’s skills or the patient’s compliance, the analysis of objective surrogate markers is needed. qMRI is a reliable imaging biomarker that can detect even subclinical changes in stable or even improved patients [35, 48, 50, 56]. The muscle FF of the thigh was shown to correlate with disease progression, to predict loss of ambulation and to have strong consistency, especially with the D1 domain of MFM [35, 48, 50, 51, 56]. According to as yet unpublished data from our group, when using the FF of the thigh muscles, a sample size of 6 is sufficient to show disease stabilization due to an active treatment over an observation time of 12 months. In comparison, even the most sensitive D1 domain of MFM requires a sample size of 12, and the total MFM score of 72 to see a treatment effect in 1 year, emphasizing the combination use of both clinical and radiologic outcome measures. Besides efficacy data, regular safety assessments including vital parameters, laboratory values, ophthalmological examination, and evaluation of cognitive function are also included to give useful information about potential risks of the treatment.

Our study not only aims to show safety and efficacy of tamoxifen in children with DMD, but also to gain a better understanding of the mechanism of action of tamoxifen. For this purpose, biomarkers of tamoxifen metabolism and muscle degeneration will be measured throughout the study. The deeper insight into how tamoxifen acts in the muscle might encourage the use of this drug in a broader spectrum of neuromuscular disorders.

## Trial status

This is protocol version 8.0 from 8 April 2019. Enrolment in this trial started in June 2018 and is expected to be completed with the OLE phase by the end of 2020. The last visit of the last patient is planned to take place in December 2021.

## Supplementary information

**Additional file 1.** SPIRIT 2013 checklist: recommended items to address in a clinical trial protocol and related documents.

## Data Availability

Not applicable.

## Abbreviations

10MWT: 10-meter walk/run test
6MWT: 6-min walk test
AFP: Alpha-fetoprotein
ANCOVA: Analysis of covariance
AP: Alkaline phosphatase
CK: Creatine kinase
CRO: Clinical Research Organization
CTU: Clinical trial unit
DEXA: Dual-energy x-ray absorptiometry
DAPC: Dystrophin-associated protein complex
DMD: Duchenne muscular dystrophy
EMA: European Medicines Agency
FF: Fat fraction
FSH: Follicle-stimulating hormone
ICH-GCP: International Conference on Harmonization Good Clinical Practice
IGF: Insulin growth factor
IL: Interleukin
LH: Luteinizing hormone
MFM: Motor function measure
MMP-2: Matrix metalloproteinase
NSAA: North Star Ambulatory Assessment
OLE: Open-label extension
PARS-III: Personal-Adjustment and Role Skills Scale
PUL: Proximal upper limb function
qMRI: Quantitative magnetic resonance imaging
QMT: Quantitative muscle testing
RCPM: Raven’s Coloured Progressive Matrices
SHBG: Sex hormone binding globulin
T2: T2 relaxation time
TFT: Timed function tests
TGF: Transforming growth factor
TNF: Tumor necrosis factor

## References

[1] Theadom A, Rodrigues M, Roxburgh R, Balalla S, Higgins C, Bhattacharjee R (2014). Prevalence of muscular dystrophies: a systematic literature review. Neuroepidemiology.

[2] Ryder S, Leadley RM, Armstrong N, Westwood M, de Kock S, Butt T (2017). The burden, epidemiology, costs and treatment for Duchenne muscular dystrophy: an evidence review. Orphanet J Rare Dis.

[3] Muntoni F, Torelli S, Ferlini A (2003). Dystrophin and mutations: one gene, several proteins, multiple phenotypes. Lancet Neurol.

[4] Blake DJ, Weir A, Newey SE, Davies KE (2002). Function and genetics of dystrophin and dystrophin-related proteins in muscle. Physiol Rev.

[5] Allen DG, Whitehead NP, Froehner SC (2016). Absence of dystrophin disrupts skeletal muscle signaling: roles of Ca2+, reactive oxygen species, and nitric oxide in the development of muscular dystrophy. Physiol Rev.

[6] Ruegg UT (2013). Pharmacological prospects in the treatment of Duchenne muscular dystrophy. Curr Opin Neurol.

[7] Gailly P (2002). New aspects of calcium signaling in skeletal muscle cells: implications in Duchenne muscular dystrophy. Biochim Biophys Acta.

[8] Vallejo-Illarramendi A, Toral-Ojeda I, Aldanondo G, López de Munain A (2014). Dysregulation of calcium homeostasis in muscular dystrophies. Expert Rev Mol Med.

[9] Bodensteiner JB, Engel AG (1978). Intracellular calcium accumulation in Duchenne dystrophy and other myopathies: a study of 567,000 muscle fibers in 114 biopsies. Neurology.

[10] Jackson MJ, Jones DA, Edwards RH (1985). Measurements of calcium and other elements in muscle biopsy samples from patients with Duchenne muscular dystrophy. Clin Chim Acta.

[11] Turner PR, Westwood T, Regen CM, Steinhardt RA (1988). Increased protein degradation results from elevated free calcium levels found in muscle from mdx mice. Nature.

[12] Tutdibi O, Brinkmeier H, Rüdel R, Föhr KJ (1999). Increased calcium entry into dystrophin-deficient muscle fibres of MDX and ADR-MDX mice is reduced by ion channel blockers. J Physiol.

[13] Millay DP, Goonasekera SA, Sargent MA, Maillet M, Aronow BJ, Molkentin JD (2009). Calcium influx is sufficient to induce muscular dystrophy through a TRPC-dependent mechanism. Proc Natl Acad Sci U S A.

[14] Nowak KJ, Davies KE (2004). Duchenne muscular dystrophy and dystrophin: pathogenesis and opportunities for treatment. EMBO Rep.

[15] Peddi PF (2018). Hormone receptor positive breast cancer: state of the art. Curr Opin Obstet Gynecol.

[16] Custódio JB, Dinis TC, Almeida LM, Madeira VM (1994). Tamoxifen and hydroxytamoxifen as intramembraneous inhibitors of lipid peroxidation. Evidence for peroxyl radical scavenging activity. Biochem Pharmacol.

[17] Custodio JB, Moreno AJ, Wallace KB (1998). Tamoxifen inhibits induction of the mitochondrial permeability transition by Ca2+ and inorganic phosphate. Toxicol Appl Pharmacol.

[18] Dodds ML, Kargacin ME, Kargacin GJ (2001). Effects of anti-oestrogens and beta-estradiol on calcium uptake by cardiac sarcoplasmic reticulum. Br J Pharmacol.

[19] Vogel VG, Costantino JP, Wickerham DL, Cronin WM, Cecchini RS, Atkins JN, National Surgical Adjuvant Breast and Bowel Project (NSABP) (2006). Effects of tamoxifen vs raloxifene on the risk of developing invasive breast cancer and other disease outcomes: the NSABP Study of Tamoxifen and Raloxifene (STAR) P-2 trial. JAMA.

[20] Kreher NC, Eugster EA, Shankar RR (2005). The use of tamoxifen to improve height potential in short pubertal boys. Pediatrics.

[21] Dorchies OM, Reutenauer-Patte J, Dahmane E, Ismail HM, Petermann O, Patthey-Vuadens O (2013). The anticancer drug tamoxifen counteracts the pathology in a mouse model of Duchenne muscular dystrophy. Am J Pathol.

[22] Gayi E, Neff LA, Ismail HM, Ruegg UT, Scapozza L, Dorchies OM (2018). Repurposing the selective oestrogen receptor modulator tamoxifen for the treatment of Duchenne muscular dystrophy. Chimia (Aarau).

[23] Gayi E, Neff LA, Massana Muñoz X, Ismail HM, Sierra M, Mercier T (2018). Tamoxifen prolongs survival and alleviates symptoms in mice with fatal X-linked myotubular myopathy. Nat Commun.

[24] Maani N, Sabha N, Rezai K, Ramani A, Groom L, Eltayeb N (2018). Tamoxifen therapy in a murine model of myotubular myopathy. Nat Commun.

[25] Walter AW (2000). Tamoxifen and carboplatin for children with low-grade gliomas: a pilot study at St. Jude Children’s Research Hospital. J Pediatr Hematol Oncol.

[26] Lawrence SE, Arnold Faught K, Vethamuthu J, Lawson ML (2004). Beneficial effects of raloxifene and tamoxifen in the treatment of pubertal gynecomastia. J Pediatr.

[27] de Aquino Gorayeb MM, Aisen S, Nadalin W, Panico Gorayeb R, de Andrade Carvalho H (2006). Treatment of childhood diffuse brain stem tumors: comparison of results in different treatment modalities. Clin Transl Oncol.

[28] Skapek SX (2013). Safety and efficacy of high-dose tamoxifen and sulindac for desmoid tumor in children: results of a Children’s Oncology Group (COG) phase II study. Pediatr Blood Cancer.

[29] Bérard C, Payan C, Hodgkinson I, Fermanian J, MFM Collaborative Study Group (2005). A motor function measure for neuromuscular diseases. Construction and validation study. Neuromuscul Disord.

[30] Bérard C, Vuillerot C, Girardot F, Payan C, the MFM study group (2017). MFM User’s Manual and score sheet.

[31] Nagy S, Schmidt S, Hafner P, Klein A, Rubino-Nacht D, Gocheva V, et al. Measurements of motor function and other clinical outcome parameters in ambulant children with Duchenne muscular dystrophy. J Vis Exp. 2019;(143). 10.3791/58784. 10.3791/58784 30688316

[32] Schreiber A, Brochard S, Rippert P, Fontaine-Carbonnel S, Payan C, Poirot I, MFM DMD Corticosteroids Group (2018). Corticosteroids in Duchenne muscular dystrophy: impact on the motor function measure sensitivity to change and implications for clinical trials. Dev Med Child Neurol.

[33] Vuillerot C, Girardot F, Payan C, Fermanian J, Iwaz J, De Lattre C (2010). Monitoring changes and predicting loss of ambulation in Duchenne muscular dystrophy with the motor function measure. Dev Med Child Neurol.

[34] Vuillerot C, Payan C, Girardot F, Fermanian J, Iwaz J, Bérard C, Group MS (2012). Responsiveness of the motor function measure in neuromuscular diseases. Arch Phys Med Rehabil.

[35] Bonati U, Hafner P, Schädelin S, Schmid M, Naduvilekoot Devasia A, Schroeder J (2015). Quantitative muscle MRI: a powerful surrogate outcome measure in Duchenne muscular dystrophy. Neuromuscul Disord.

[36] Mazzone E, Martinelli D, Berardinelli A, Messina S, D'Amico A, Vasco G (2010). North Star Ambulatory Assessment, 6-minute walk test and timed items in ambulant boys with Duchenne muscular dystrophy. Neuromuscul Disord.

[37] Mazzone E, Messina S, Vasco G, Main M, Eagle M, D'Amico A (2009). Reliability of the North Star Ambulatory Assessment in a multicentric setting. Neuromuscul Disord.

[38] De Sanctis R, Pane M, Sivo S, Ricotti V, Baranello G, Frosini S (2015). Suitability of North Star Ambulatory Assessment in young boys with Duchenne muscular dystrophy. Neuromuscul Disord.

[39] Mercuri E, Coratti G, Messina S, Ricotti V, Baranello G, D'Amico A (2016). Revised North Star Ambulatory Assessment for young boys with Duchenne muscular dystrophy. PLoS One.

[40] Pane M, Mazzone ES, Sivo S, Sormani MP, Messina S, D'Amico A (2014). Long term natural history data in ambulant boys with Duchenne muscular dystrophy: 36-month changes. PLoS One.

[41] McDonald CM, Henricson EK, Han JJ, Abresch RT, Nicorici A, Elfring GL (2010). The 6-minute walk test as a new outcome measure in Duchenne muscular dystrophy. Muscle Nerve.

[42] McDonald CM, Henricson EK, Abresch RT, Florence JM, Eagle M, Gappmaier E (2013). The 6-minute walk test and other endpoints in Duchenne muscular dystrophy: longitudinal natural history observations over 48 weeks from a multicenter study. Muscle Nerve.

[43] McDonald CM, Henricson EK, Abresch RT, Florence J, Eagle M, Gappmaier E (2013). The 6-minute walk test and other clinical endpoints in Duchenne muscular dystrophy: reliability, concurrent validity, and minimal clinically important differences from a multicenter study. Muscle Nerve.

[44] Mazzone ES, Pane M, Sormani MP, Scalise R, Berardinelli A, Messina S (2013). 24 month longitudinal data in ambulant boys with Duchenne muscular dystrophy. PLoS One.

[45] Pane M, Mazzone ES, Fanelli L, De Sanctis R, Bianco F, Sivo S (2014). Reliability of the performance of upper limb assessment in Duchenne muscular dystrophy. Neuromuscul Disord.

[46] Pane M, Coratti G, Brogna C, Mazzone ES, Mayhew A, Fanelli L (2018). Upper limb function in Duchenne muscular dystrophy: 24 month longitudinal data. PLoS One.

[47] Hébert LJ, Remec JF, Saulnier J, Vial C, Puymirat J (2010). The use of muscle strength assessed with handheld dynamometers as a non-invasive biological marker in myotonic dystrophy type 1 patients: a multicenter study. BMC Musculoskelet Disord.

[48] Godi C, Ambrosi A, Nicastro F, Previtali SC, Santarosa C, Napolitano S (2016). Longitudinal MRI quantification of muscle degeneration in Duchenne muscular dystrophy. Ann Clin Transl Neurol.

[49] Morrow JM, Sinclair CD, Fischmann A, Machado PM, Reilly MM, Yousry TA (2016). MRI biomarker assessment of neuromuscular disease progression: a prospective observational cohort study. Lancet Neurol.

[50] Willcocks RJ, Rooney WD, Triplett WT, Forbes SC, Lott DJ, Senesac CR (2016). Multicenter prospective longitudinal study of magnetic resonance biomarkers in a large Duchenne muscular dystrophy cohort. Ann Neurol.

[51] Schmidt S, Hafner P, Klein A, Rubino-Nacht D, Gocheva V, Schroeder J (2018). Timed function tests, motor function measure, and quantitative thigh muscle MRI in ambulant children with Duchenne muscular dystrophy: a cross-sectional analysis. Neuromuscul Disord.

[52] Hafner P, Bonati U, Rubino D, Gocheva V, Zumbrunn T, Gueven N, Fischer D (2016). Treatment with L-citrulline and metformin in Duchenne muscular dystrophy: study protocol for a single-centre, randomised, placebo-controlled trial. Trials.

[53] Manzur AY, Kuntzer T, Pike M, Swan A. Glucocorticoid corticosteroids for Duchenne muscular dystrophy. Cochrane Database Syst Rev. 2008;23(1):CD003725 Update in: Cochrane Database Syst Rev. 2016;(5):CD003725. 10.1002/14651858.CD003725.pub3 18254031

[54] Hendriksen JG, Poysky JT, Schrans DG, Schouten EG, Aldenkamp AP, Vles JS (2009). Psychosocial adjustment in males with Duchenne muscular dystrophy: psychometric properties and clinical utility of a parent-report questionnaire. J Pediatr Psychol.

[55] Wicksell RK, Kihlgren M, Melin L, Eeg-Olofsson O (2004). Specific cognitive deficits are common in children with Duchenne muscular dystrophy. Dev Med Child Neurol.

[56] Fischmann A, Hafner P, Gloor M, Schmid M, Klein A, Pohlman U (2013). Quantitative MRI and loss of free ambulation in Duchenne muscular dystrophy. J Neurol.

